# The Convergence Study of the Homotopy Analysis Method for Solving Nonlinear Volterra-Fredholm Integrodifferential Equations

**DOI:** 10.1155/2014/465951

**Published:** 2014-01-12

**Authors:** Behzad Ghanbari

**Affiliations:** Department of Basic Sciences, Kermanshah University of Technology, P.O. Box 63766-67178, Kermanshah, Iran

## Abstract

We aim to study the convergence of the homotopy analysis method (HAM in short) for solving special nonlinear Volterra-Fredholm integrodifferential equations. The sufficient condition for the convergence of the method is briefly addressed. Some illustrative examples are also presented to demonstrate the validity and applicability of the technique. Comparison of the obtained results HAM with exact solution shows that the method is reliable and capable of providing analytic treatment for solving such equations.

## 1. Introduction

In recent years, there has been a growing interest in the nonlinear Volterra–Fredholm integrodifferential equations, NVFID, in short, which are a combination of differential and Fredholm-Volterra integral equations. These equations occur frequently as a model in mathematical biology and the physical sciences. Volterra–Fredholm integrodifferential equations is sometimes difficult to solve analytically. Therefore, finding either the analytical approximation or numerical solution of such equations is of great interest. The interested reader can refer to [1–11] (and the references therein) for more research works.

We aim in this work to study the convergence of the homotopy analysis method, for solving NVFID equations, of the form:

(1) u′(x)+q(x)u(x)+λ1∫01k1(x,t)(u(t))pdt   +λ2∫0xk2(x,t)(u(t))qdt=f(x), p,q∈N,u(0)=u0, x∈[0,1],

where *μ* is a real number, the kernel *k*_1_(*x*, *t*), *k*_2_(*x*, *t*) are known continuous functions over [*a*, *b*] × [*a*, *b*], and *f*(*x*), *q*(*x*) are given continuous function defined over [*a*, *b*].

The homotopy analysis method (HAM) [12] has been proved to be one of the useful techniques to solve numerous linear and nonlinear functional equations [13–17].

As mentioned in [13, 14], unlike all previous analytic techniques, the homotopy analysis method provides great freedom to express solutions of a given nonlinear problem by means of different base functions. Also this method provides a way to adjust and control the convergence region and the rate of convergence of solution series, by introducing the auxiliary parameter *ℏ*.

By properly choosing the base functions, initial approximations, auxiliary linear operators, and auxiliary parameter *ℏ*, HAM gives rapidly convergent successive approximations of the exact solution. A systematic description of this analytic technique, for nonlinear problems, can be found in [13].

This paper is organized as follows. In Section 2, a short description of the basic ideas of the homotopy analysis method will be stated. In Section 3, the homotopy analysis method is applied to construct approximate solution of (1).  Section 4 is devoted to the convergence analysis of the method. In Section 5, our numerical findings are reported and demonstrate the accuracy of the proposed scheme, by considering three numerical examples. Finally, conclusions are stated in the last section.

## 2. Basic Idea of HAM 

Let us assume the following nonlinear differential:

(2) $$\begin{array}{c} N\left[u\left(\tau \right)\right]=0, \end{array}$$

where *N* is a nonlinear operator, *τ* is an independent variable, and *u*(*τ*) is the solution of the equation.

By utilizing the concept of homotopy in topology, Liao [13] constructs the so-called zero-order deformation equation

(3) $$\begin{array}{c} \left(1-q\right)L\left[\Phi \left(x;q\right)-u_{0}\left(x\right)\right]-\hbar qN\left[\Phi \left(x;q\right)\right]=0, \end{array}$$

where *q* ∈ [0,1] is the embedding parameter, *ℏ* ≠ 0 is a nonzero auxiliary parameter, *L* is an auxiliary linear operator, *u*_0_(*x*) is an initial guess of *u*(*x*), and Φ(*x*; *q*) is an unknown function, respectively.

In the view of HAM the solution of original equation is assumed to be expanded in terms of embedding parameter *q* as

(4) $$\begin{array}{c} \Phi \left(x;q\right)=u_{0}\left(x\right)+\sum_{i=0}^{\infty }u_{i}\left(x\right)q^{i}, \end{array}$$

where *u*_*i*_(*x*)'s are obtained as follows.

Appling recently proposed “*m*th-order homotopy-derivative operator” [14]:

(5) $$\begin{array}{c} D_{m}\left(\phi \right)={\frac{1}{m!}\frac{\partial^{m}\phi }{\partial q^{m}}|}_{q=0} \end{array}$$

to both sides of (3), one reads

(6) $$\begin{array}{c} L\left[u_{m}\left(x\right)-\chi_{m}u_{m-1}\left(x\right)\right]=hR_{m-1}\left(x\right), \end{array}$$

subject to initial condition

(7) $$\begin{array}{c} u_{m}\left(0\right)=0, \end{array}$$

where

(8) Rm−1(x)=Dm−1(N[u(x;q)])=1(m−1)!∂m−1N[u(x;q)]∂qm−1|q=0,χm={1,m>1,0,m≤1.

In this way *u*_*m*_'s, for *m* ≥ 1, are dependent upon not only *x* but also the auxiliary parameter *ℏ*.

As proved in [13], whenever (4) is a convergent series at *q* = 1, its limit must satisfy the original equation *N*[*u*(*τ*)] = 0. To find a proper convergence-control parameter *ℏ*, to get a convergent series solution or to accelerate its convergence rate, there is a classic way of plotting the so-called ‘‘*ℏ*-curves” or ‘‘curves for convergence-control parameter”. Such a region can be found, although approximately, by plotting the curves of these unknown quantities versus *ℏ*.

However, it is a pity that curves for convergence-control parameter (i.e., *ℏ*-curves) give us only a graphical region and cannot tell us which value of *ℏ*_0_ ∈ *R*_*ℏ*_ gives the fastest convergent series. Moreover, recently in [18] a misinterpreted usage of *ℏ*-curves has been reported.

## 3. HAM for NVFID

To investigate the exact solution of (1), by means of the homotopy analysis method, let us consider the nonlinear operator

(9) N[Φ(x;q)]=∂Φ(x;q)∂x+q(x)Φ(x;q)+λ1∫01k1(x,t)(Φ(x;q))pdt  +λ2∫0xk2(x,t)(Φ(x;q))qdt−f(x),

and linear operator

(10) $$\begin{array}{c} L\phi =\frac{d\phi }{dx}, \end{array}$$

with the following property:

(11) $$\begin{array}{c} L\left[c\right]=0, \end{array}$$

where *c* is constant.

The initial guess *u*_0_(*x*) is chosen such that it satisfies the initial condition of problem; that is,

(12) $$\begin{array}{c} u_{0}\left(0\right)=u\left(0\right)=u_{0}. \end{array}$$

from (8) and by using (9), we have

(13) Rm[ϕ→m−1(x)]=∂ϕm−1∂x+q(x)ϕm−1−(1−χm)f(x) +λ1∫01k1(x,t)[∑r1=0m−1ϕm−1−r1(x)×∑r2=0r1ϕr1−r2(x)∑r3=0r2ϕr2−r3(x)⋯∑rp−2=0rp−3ϕrp−3−rp−2(x)×∑rp−1=0rp−2ϕrp−2−rp−1(x)ϕrp−1(x)]dt +λ2∫0xk2(x,t)[∑r1=0m−1ϕm−1−r1(x)∑r2=0r1ϕr1−r2(x)×∑r3=0r2ϕr2−r3(x)⋯∑rp−2=0rp−3ϕrq−3−rp−2(x)×∑rp−1=0rp−2ϕrq−2−rp−1(x)ϕrq−1(x)]dt.

From (13), it should be noted that the right-hand side of (6) is only dependent upon ${\vec{\phi }}_{m-1}$. Thus, *ϕ*_0_(*r*, *t*), *ϕ*_1_(*r*, *t*), *ϕ*_2_(*r*, *t*),…, will be computed recursively, by means of solving the linear high-order deformation equation (6) subject to (7). So, the *m*th-order approximation of *u*(*x*) is given by

(14) $$\begin{array}{c} u_{m}\left(x\right)=\sum_{k=0}^{m}\phi_{k}\left(x\right). \end{array}$$

## 4. Convergence Analysis

In this section, some theorems and conditions in the framework of convergence of the homotopy analysis method are stated.

**Theorem 1**

Whereas the series

(15) $$\begin{array}{c} \phi_{0}\left(x\right)+\sum_{m=1}^{+\infty }\phi_{m}\left(x\right) \end{array}$$

converges, where *ϕ*_*m*_(*x*)'s are resulted from (6), (7), and (13), the limit of the series is an exact solution of (1).

**Proof**

Since, by hypothesis, the series is convergent, it holds

(16) $$\begin{array}{c} s\left(x\right)=\sum_{m=0}^{+\infty }\phi_{m}\left(x\right). \end{array}$$

So, the necessary condition for the convergence of the series is valid; that is,

(17) $$\begin{array}{c} \underset{m\to \infty }{\lim}\phi_{m}\left(x\right)=0. \end{array}$$

Using (6) and (17), we have

(18) ℏH(x)∑m=1+∞Rm[ϕ→m−1(x)] =lim⁡n→∞∑m=1nL[ϕm(x)−χmϕm−1(x)] =L{lim⁡n→∞∑m=1n[ϕm(x)−χmϕm−1(x)]} =L{lim⁡n→∞ϕn(x)} =0.

Since *ℏ* ≠ 0, we must have

(19) $$\begin{array}{c} \sum_{m=1}^{+\infty }R_{m}\left[{\vec{\phi }}_{m-1}\left(x\right)\right]=0. \end{array}$$

On the other hand, we have

(20) ∑m=1+∞Rm[ϕ→m−1(x)]   =∑m=1n[∂ϕm−1∂x+q(x)ϕm−1−(1−χm)f(x)  +λ1  ∫01k1(x,t)[∑r1=0m−1ϕm−1−r1(x)∑r2=0r1ϕr1−r2(x)×∑r3=0r2ϕr2−r3(x)…∑rp−2=0rp−3ϕrp−3−rp−2(x)×∑rp−1=0rp−2ϕrp−2−rp−1(x)×ϕrp−1(x)]dt+λ2∫0xk2(x,t)[∑r1=0m−1ϕm−1−r1(x)∑r2=0r1ϕr1−r2(x)×∑r3=0r2ϕr2−r3(x)…∑rp−2=0rp−3ϕrq−3−rp−2(x)×∑rp−1=0rp−2ϕrq−2−rp−1(x)×ϕrq−1(x)]dt] =∑m=0∞ϕm′(x)+q(x)∑m=0∞ϕm(x)−f(x)  +λ1∑m=1∞{∫01k1(x,t)×[∑r1=0m−1ϕm−1−r1(x)∑r2=0r1ϕr1−r2(x)×∑r3=0r2ϕr2−r3(x)…∑rp−2=0rp−3ϕrp−3−rp−2(x)×∑rp−1=0rp−2ϕrp−2−rp−1(x)×ϕrp−1(x)]dt}  +λ2∑m=1∞{∫0xk2(x,t)[∑r1=0m−1ϕm−1−r1(x)∑r2=0r1ϕr1−r2(x)×∑r3=0r2ϕr2−r3(x)…∑rq−2=0rq−3ϕrq−3−rp−2(x)×∑rq−1=0rq−2ϕrq−2−rq−1×(x)ϕrq−1(x)]dt} =∑m=0∞ϕm′(x)+q(x)∑m=0∞ϕm(x)−f(x)  +λ1∫01k1(x,t)[∑rp−1=0∞ϕm−1−r1(x)∑rp−2=rp−1∞ϕr1−r2(x)×∑rp−2=rp−1∞ϕr2−r3(x)…∑r2=r3∞ϕrp−3−rp−2(x)×∑r1=r2∞ϕrp−2−rp−1(x)×∑m=r1∞ϕm−r1(x)]dt  +λ2∫0xk2(x,t)[∑rq−1=0∞ϕm−1−r1(x)∑rq−2=rq−1∞ϕr1−r2(x)×∑rq−2=rq−1∞ϕr2−r3(x)…∑r2=r3∞ϕrp−3−rq−2(x)×∑r1=r2∞ϕrq−2−rq−1(x)∑m=r1∞ϕm−r1(x)]dt =∑m=0∞ϕm′(x)+q(x)∑m=0∞ϕm(x)−f(x)    +λ1∫  0  1k1(x,t)[∑i1=0∞ϕi1(x)∑i2=0∞ϕi2(x)∑i3=0∞ϕi3(x)…∑ip−2=0∞ϕip−2(x)×∑ip−1=0∞ϕip−1(x)∑ip=0∞ϕip(x)]dt  +λ2  ∫  0xk2(x,t)[∑j1=0∞ϕj1(x)∑j2=0∞ϕj2(x)×∑j3=0∞ϕj3(x)…∑jq−2=0∞ϕjq−2(x)×∑jq−1=0∞ϕjq−1(x)∑jq=0∞ϕjq(x)]dt =s′(x)+q(x)s(x)−f(x)  +  λ1∫  abk1(x,t)(s(t))pdt    +λ2∫0xk2(x,t)(s(t))qdt.  

So, from (19), we obtain

(21) s′(x)+q(x)s(x)+λ1∫01k1(x,t)(s(x))pdt  +λ2∫0xk2(x,t)(s(x))qdt=f(x).

Also, from initial conditions (7) and (12), the following holds:

(22) $$\begin{array}{c} s\left(0\right)=\sum_{i=0}^{\infty }\phi_{i}\left(0\right)=\phi_{0}\left(0\right)=u_{0}\left(0\right)=u_{0}. \end{array}$$

Since *s*(*x*) satisfies (21) and (22), we conclude that it is an exact solution of (1). This completes the proof.

**Theorem 2**

Suppose that *ℑ* ⊂ ℝ is a Banach space with a suitable norm ||·||, say ||·||_*∞*_, over which the sequence *ϕ*_*m*_(*x*) is defined for a prescribed value of *ℏ*. Assume also that the initial approximation *ϕ*_0_(*x*) remains inside the domain of the solution *u*(*x*). Taking *r* ∈ *R* as a constant, the following statements holds.

- If there exists some *r* ∈ [0,1], such that for all *k* ∈ *ℕ* we have ||*ϕ*_*k*+1_(*x*)|| ≥ *r*||*ϕ*_*k*_(*x*)||, then the series solution *u*(*x*) = ∑_*k*=0_^*∞*^ *ϕ*_*k*_(*x*)*q* ^*k*^ converges absolutely at *q* = 1, over the domain of *x*.
- If there exists some *r* > 1, such that for all *k* ∈ *ℕ* we have ||*ϕ*_*k*+1_(*x*)|| ≤ *r*||*ϕ*_*k*_(*x*)||, then the series solution *u*(*x*) = ∑_*k*=0_^*∞*^ *ϕ*_*k*_(*x*)*q* ^*k*^ diverges, at *q* = 1, over the domain of definition of *x*.

**Proof**

Indeed, this is a special case of Banach' fix point theorem, which can be found in standard texts on real analysis such as in [19].

Theorems 1 and 2 state that the homotopy series solution ∑_*k*=0_^*∞*^ *ϕ*_*k*_(*x*), of nonlinear problem (1), converges to an exact solution *u*(*x*), under the condition that ∃*γ*, 0 < *γ* < 1 such that ∀*k* ≥ *k*_0_, ||*ϕ*_*k*+1_(*x*)|| ≤ *γ*||*ϕ*_*k*_(*x*)||, for some *k*_0_ ∈ *ℕ*. In other words, if, for every *i* ≥ *k*_0_, we consider the parameters *β*_i_'s, as were defined in [20],

(23) $$\begin{array}{c} \beta_{i+1}=\{\begin{array}{cc} \frac{||\phi_{i+1}||}{||\phi_{i}||}, & ||\phi_{i}||\neq 0,\\ 0, & ||\phi_{i}||=0, \end{array} \end{array}$$

for *i* ∈ *ℕ* ∪ {0}, then the series solution∑_*k*=0_^*∞*^ *ϕ*_*k*_(*x*) of problem (1) converges to an exact solution *u*(*x*), when ∀*i* ≥ *k*_0_, 0 ≤ *β*_*i*_ < 1.

## 5. Numerical Results 

To illustrate the convergence study of HAM, two examples are presented.

**Example 1**

Let us first consider the following linear Fredholm integral equation:

(24) u′(x)+u(x)−14∫01t(u(t))3dt  +12∫0xx(u(t))2dt=f(x),

where *f*(*x*) = 2*x* + *x* ^2^ + (1/10)*x* ^6^ − (1/32), with the initial condition *u*(0) = 0. The exact solution is *u*(*x*) = *x* ^2^ [6].

To solve (24) by means of HAM and according to the initial condition, we can choose the initial guess of *u*(*x*) as follows:

(25) $$\begin{array}{c} \phi_{0}^{}=x \end{array}$$

The linear operator and the zero-order deformation equations are as defined by (10) and (3), respectively.

Therefore, the *m*th-order deformation equations (6) read as

(26) $$\begin{array}{c} \frac{d}{dx}\left[u_{m}\left(x\right)-\chi_{m}u_{m-1}\left(x\right)\right]=hR_{m-1}\left(x\right), \end{array}$$

subject to initial condition

(27) $$\begin{array}{c} u_{m}\left(0\right)=0, \end{array}$$

where

(28) Rm−1(x)=∂ϕm−1∂x+ϕm−1−(1−χm)f(x)−14∫01t[∑r1=0m−1ϕm−1−r1(x)∑r2=0r1ϕr1−r2(x)ϕr2(x)]dt+12∫0xx[∑r1=0m−1ϕm−1−r1(x)ϕr1(x)]dt.

Now, we can successively obtain the solution of the *m*th-order deformation equation (26) and (27), for *m* ≥ 1.

Table 1 shows some values of *β*_*i*_'s, defined as in (23), which were obtained from the HAM series solution (14) by using different values of *ℏ*. Form Table 1, since *β*_*i*_ < 1 for *ℏ* = −0.74, *ℏ* = −0.9, and *ℏ* = −1, we can conclude that the HAM approach converges to the exact solution for *ℏ* = −0.74, *ℏ* = −0.9, and *ℏ* = −1. Also, one can observe that *β*_*i*_'s are not less than one for *ℏ* = 0.4. So, the HAM approach may be divergent for *ℏ* = 0.4.

In Table 2 the relative errors *δ*_*n*_ of the *n* terms approximation of HAM, which is defined as

(29) $$\begin{array}{c} \delta \left(x_{j}\right)=\left|\frac{u_{\text{exact}}\left(x_{j}\right)-u_{\text{appr}}\left(x_{j}\right)}{u_{\text{exact}}\left(x_{j}\right)}\right|, \end{array}$$

for different values of *ℏ*, are presented. It is evident that the auxiliary parameter *ℏ* can also be effectively implemented to adjust and control the rate of convergence of the series solutions by HAM.

**Example 2**

Consider now the following equation [7, 11]:

(30) u′(x)−∫0xcos⁡(x−t)(u(t))2dt, =−2sin(x)−13cos⁡(x)−23cos⁡(2x),

with initial condition *u*(0) = 1. The exact solution is *u*(*x*) = cos⁡(*x*) − sin(*x*).

To obtain the approximate solution of this example, if we choose *ϕ*_0_(*x*) = 1, then the solutions of *m*th-order deformation equations can be resulted in (6) and (7), where

(31) Rm−1(x)=∂ϕm−1∂x+ϕm−1−(1−χm)×[−2sin(x)−13cos⁡(x)−23cos⁡(2x)]−∫01cos⁡(x−t)[∑r1=0m−1ϕm−1−r1(x)ϕr1(x)]dt.

Since HAM provides a family of solution expression in the auxiliary parameter *h*, the convergence region of solution series depends upon the value of *ℏ*.

Table 3 shows the computed values of *β*_*i*_'s for different values of *ℏ*, in this example. Also, for different values of *ℏ*, the relative errors of approximate analytic solutions by HAM solution are reported in Table 4.

Clearly, one can observe that the approximate solution for *ℏ* = −1, is more accurate than the approximate solutions obtained for *ℏ* = −1.1, *ℏ* = −0.9, and *ℏ* = −0.8. Therefore, it can be claimed that the auxiliary parameter *ℏ* plays an important role to adjust and control the convergence of the series solution. It can be seen from Tables 3 and 4 that, for smaller values of *β*_*i*_, our approximate solutions are in very excellent agreement with the exact solutions.

## 6. Conclusion

In this paper, we presented the application of the homotopy analysis method (HAM) for solving a special form of nonlinear Volterra-Fredholm integro-differential equation.

The sufficient condition for the convergence of the method is illustrated and then verified for two examples.

As we can see in Tables 2 and 4, HAM solutions have a good agreement with the numerical results provided that appropriate values for the convergence control parameter *ℏ* are chosen.

The ability of the HAM is mainly due to the fact that the method provides a way to ensure the convergence of series solution.

Convergence conditions in applying HAM for other equations, and systems of ordinary and partial differential equations, integral equations, and integro-differential equations are also under study in our research team.

## Figures and Tables

**Table 1:** Numerical values of *β*_*i*_'s for different values of ℏ in Example 1.

	ℏ = −1	ℏ = −0.9	ℏ = −0.74	ℏ = 0.4
*β*_1_	0.675127819	0.558404720	0.371647762	1.53698244
*β*_2_	0.250131472	0.119092776	0.360689399	1.540085431
*β*_3_	0.466457456	1.052930969	0.097317362	1.543843327
*β*_4_	0.971262707	0.366763445	0.669779432	1.548761762
*β*_5_	0.378085555	0.182601166	0.068939396	1.555540384
*β*_6_	0.305191320	1.321695721	0.700358533	1.565066508

**Table 2:** Comparison of relative errors *δ*(*x*_*j*_) for Example 1.

	ℏ = −1	ℏ = −0.9	ℏ = −0.74	ℏ = 0.4
*x*_1_ = 0.1	2.12*E* − 2	1.46*E* − 3	7.53*E* − 6	93.053
*x*_2_ = 0.2	1.14*E* − 2	9.28*E* − 4	7.14*E* − 5	45.719
*x*_3_ = 0.3	8.05*E* − 3	7.65*E* − 4	2.69*E* − 5	29.642
*x*_4_ = 0.4	6.34*E* − 3	6.98*E* − 4	1.80*E* − 5	21.377
*x*_5_ = 0.5	5.29*E* − 3	6.70*E* − 4	4.92*E* − 5	16.237
*x*_6_ = 0.6	4.55*E* − 3	6.64*E* − 4	6.69*E* − 5	12.660
*x*_7_ = 0.7	3.96*E* − 3	6.72*E* − 4	7.45*E* − 5	9.975
*x*_8_ = 0.8	3.45*E* − 3	6.88*E* − 4	7.59*E* − 5	7.846
*x*_9_ = 0.9	2.96*E* − 3	7.06*E* − 4	7.36*E* − 5	6.080
*x*_10_ = 1.0	2.45*E* − 3	7.23*E* − 4	6.90*E* − 5	4.555

**Table 3:** Numerical values of *β*_*i*_'s for different values of ℏ in Example 2.

	ℏ = −1.25	ℏ = −1.15	ℏ = −1	ℏ = −0.9
*β*_1_	0.2161299265	0.2360588266	0.335703327	0.4021329943
*β*_2_	0.9046884934	0.7386201225	0.182536164	0.0517028491
*β*_3_	0.1557524739	0.0828773249	0.015625179	1.2627041992
*β*_4_	0.3573468358	0.82893171716	0.342118434	0.3785790309
*β*_5_	0.5384820933	0.3132925258	0.4796520139	0.1375116383
*β*_6_	0.1126211304	0.0312756855	0.0847887058	0.1544221928

**Table 4:** Comparison of relative errors *δ*(*x*_*j*_) for Example 2.

	ℏ = −1.25	ℏ = −1.15	ℏ = −1	ℏ = −0.9
*x*_1_ = 0.1	6.14*E* − 6	1.93*E* − 7	6.47*E* − 8	2.01*E* − 8
*x*_2_ = 0.2	4.74*E* − 6	1.35*E* − 7	1.27*E* − 9	1.27*E* − 8
*x*_3_ = 0.3	2.14*E* − 5	1.57*E* − 6	1.51*E* − 7	1.36*E* − 7
*x*_4_ = 0.4	9.40*E* − 5	3.60*E* − 6	3.76*E* − 7	5.25*E* − 7
*x*_5_ = 0.5	2.06*E* − 4	3.444*E* − 7	2.51*E* − 7	2.29*E* − 6
*x*_6_ = 0.6	2.28*E* − 4	3.07*E* − 5	4.06*E* − 7	3.98*E* − 6
*x*_7_ = 0.7	7.46*E* − 4	1.73*E* − 4	9.09*E* − 6	1.59*E* − 5
*x*_8_ = 0.8	1.97*E* − 2	9.06*E* − 4	2.84*E* − 6	7.76*E* − 4
*x*_9_ = 0.9	4.95*E* − 3	3.26*E* − 4	1.42*E* − 5	3.67*E* − 4
*x*_10_ = 1.0	2.99*E* − 3	9.76*E* − 4	2.61*E* − 5	5.65*E* − 4

## References

[1] Wazwaz A-M (2010). The combined Laplace transform-Adomian decomposition method for handling nonlinear Volterra integro-differential equations. *Applied Mathematics and Computation*.

[2] Babolian E, Masouri Z, Hatamzadeh-Varmazyar S (2009). Numerical solution of nonlinear Volterra-Fredholm integro-differential equations via direct method using triangular functions. *Computers and Mathematics with Applications*.

[3] Biazar J, Ghazvini H (2008). Numerical solution for special non-linear Fredholm integral equation by HPM. *Applied Mathematics and Computation*.

[4] Biazar J, Gholami Porshokouhi M, Ghanbari B, Gholami Porshokouhi M (2011). Numerical solution of functional integral equations by the variational iteration method. *Journal of Computational and Applied Mathematics*.

[5] Yildirim A (2008). Solution of BVPs for fourth-order integro-differential equations by using homotopy perturbation method. *Computers and Mathematics with Applications*.

[6] Babolian E, Masouri Z, Hatamzadeh-Varmazyar S (2009). Numerical solution of nonlinear Volterra-Fredholm integro-differential equations via direct method using triangular functions. *Computers and Mathematics with Applications*.

[7] Maleknejad K, Mahmoudi Y (2003). Taylor polynomial solution of high-order nonlinear Volterra-Fredholm integro-differential equations. *Applied Mathematics and Computation*.

[8] Maleknejad K, Kajani MT (2004). Solving linear integro-differential equation system by Galerkin methods with hybrid functions. *Applied Mathematics and Computation*.

[9] Maleknejad K, Mahmoudi Y (2003). Taylor polynomial solution of high-order nonlinear Volterra-Fredholm integro-differential equations. *Applied Mathematics and Computation*.

[10] Babolian E, Masouri Z, Hatamzadeh-Varmazyar S (2008). New direct method to solve nonlinear Volterra-Fredholm integral and integro differential equation using operational matrix with Block-pulse functions. *Progress in Electromagnetics Research*.

[11] Maleknejad K, Basirat B, Hashemizadeh E (2011). Hybrid Legendre polynomials and Block-Pulse functions approach for nonlinear VolterraFredholm integro-differential equations. *Computers and Mathematics with Applications*.

[12] Liao SJ (1992). *Proposed homotopy analysis techniques for the solution of non-linear problem [Ph.D. dissertation]*.

[13] Liao SJ (2003). *Beyond Perturbation: Introduction to HAM*.

[14] Liao SJ (2009). Notes on the homotopy analysis method: some definitions and theorems. *Communications in Nonlinear Science and Numerical Simulation*.

[15] Ghanbari B, Rada L, Chen K A restarted iterative homotopy analysis method for two nonlinear models from image processing. *International Journal of Computer Mathematics*.

[16] Biazar J, Ghanbari B (2012). The homotopy perturbation method for solving neutral functional-differential equations with proportional delays. *Journal of King Saud University*.

[17] Biazar J, Ghanbari B (2012). HAM solution of some initial value problems arising in heat radiation equations. *Journal of King Saud University*.

[18] Mehmood A, Munawar S, Ali A (2010). Comments to: “Homotopy analysis method for solving the MHD flow over a non-linear stretching sheet (Communications in Nonlinear Science and Numerical Simulation, vol. 14, pp. 2653–2663, 2009)”. *Communications in Nonlinear Science and Numerical Simulation*.

[19] Rudin W (1953). *Principles of Mathematical Analysis*.

[20] Odibat ZM (2010). A study on the convergence of homotopy analysis method. *Applied Mathematics and Computation*.

